# Customer Engagement in Multi-Sensory Virtual Reality Advertising: The Effect of Sound and Scent Congruence

**DOI:** 10.3389/fpsyg.2022.747456

**Published:** 2022-03-21

**Authors:** Malaika Brengman, Kim Willems, Laurens De Gauquier

**Affiliations:** ^1^Marketing & Consumer Behavior Cluster, Business Department, Faculty of Social Sciences & Solvay Business School, Vrije Universiteit Brussel, Brussel, Belgium; ^2^Marketing & Strategy Department, Faculty of Business Economics, Hasselt University, Hasselt, Belgium

**Keywords:** virtual reality, sensory experience, scent, sound, telepresence, vividness, sensory congruence, customer engagement

## Abstract

Despite the power of VR in immersing viewers in an experience, it generally only targets viewers via *visual* and *auditory* cues. Human beings use more senses to gather information, so expectedly, the full potential of this medium is currently not yet tapped. This study contributes in answering two research questions: (1) How can conventional VR ads be enriched by also addressing the *forgotten sense of smell*?; and (2) Does doing so indeed instill more engaging experiences? A 2 × 3 between-subjects study (*n* = 235) is conducted, whereby an existing branded VR commercial (Boursin Sensorium Experience) is augmented with “sound” (on/off) and (congruent/incongruent/no) “scents.” The power of these sensory augmentations is evaluated by inspecting emotional, cognitive and conative dimensions of *customer engagement*. The results identify *product-scent congruence (with sound)* as a deal-maker, albeit product-scent incongruence is not necessarily a deal-breaker. The article concludes with further research avenues and a translation into managerial implications.

## Introduction

According to the Marketing Science Institute (Marketing Science Institute [MSI], 2010), firms increasingly consider non-transactional activities as a route for creating, building and enhancing customer-firm relationships. Since its first conception about a decade ago, customer engagement (CE) has steadily evolved into becoming a cornerstone concept in marketing. Most conceptualizations in the academic and business practice literature define CE as a multi-dimensional phenomenon, consisting of a combination of cognitive aspects (e.g., being interested in a company’s activities), emotional aspects (e.g., feeling positive about a company’s activities), and/or behavioral aspects (e.g., intentions to purchase) (Brodie et al., 2011; Dijkmans et al., 2015). CE as such goes beyond pure transactions, and incorporates both psychological and behavioral dimensions (e.g., Patterson et al., 2006; Brodie et al., 2011; Vivek et al., 2012; So et al., 2014).

Facing challenges of media fragmentation and information overload, as an increasing number of brands compete for the same consumer mind space, marketeers realize the potential of trying out new media, such as Virtual Reality (VR), to engage consumers (Hollebeek et al., 2019; Violante et al., 2019; Willems et al., 2019). While VR has found its origins in non-marketing related domains, such as (military) training, health care and gaming, it is steadily being considered as a tool for successfully reaching out to consumers as well (cf. e.g., JBR special issue Vol. 100; Boyd and Koles, 2019). However, while in marketing practice, VR is increasingly finding its way to adoption, scholarly research on *how to use this medium*, is still relatively limited (Loureiro et al., 2019). With head-mounted VR headsets becoming ever more available at democratic prices in consumer markets, the opportunities to enhance the customer journey with VR are vast, yet still largely unexplored (Hollebeek et al., 2020). Despite the technological advances, empirical studies to understand *how VR influences consumer behavior* have also been limited to date (Loureiro et al., 2021). In line with Petit et al. (2019) who call for further research in order to create more enjoyable and informative multisensory experiences for consumers by means of sensory enabling technologies, Loureiro et al. (2021) call explicitly to examine other sensory stimuli than visual and auditory in VR experiences. Especially addressing the sense of smell in VR settings seems promising for commercial applications (Flavian et al., 2021; Loureiro et al., 2021) as it can enhance the experience in multisensory digital environments (Raisamo et al., 2019). However, there is a lack of studies analyzing the integration of scents into VR experiences and their impact on consumer behavior (Serrano et al., 2013; Roschk and Hosseinpour, 2020; Flavian et al., 2021).

The present study will therefore start to address these calls by focusing on the role of multi-sensory VR for brand communications. Acknowledging VR’s power of immersing viewers in an experience, and of providing sensory cues beyond visual and auditory stimuli, we want to tap further into the potential of this medium to engage consumers. The literature on sensory marketing is quite vast, showing many promising results of investing in appealing to consumers’ senses. Petit et al. (2019) call explicitly for future research to better understand the persuasive power of sensory enabling technologies and how they can enhance the consumer experience. This study contributes in building knowledge on how VR, and particularly the sensory-enrichment of a transformational VR brand ad, can instill more engaging experiences. Our findings aim to contribute to a better understanding of how multiple sensory inputs can deliver holistic digital experiences which foster affective and behavioral reactions (Nibbe and Orth, 2017). The focus hereby is on the sense of audition and the sense of smell. The choice for the former is informed by the feasibility of enabling sound in conventional VR experiences. The attention for the latter is based on the fact that olfaction is the most widely studied “forgotten” sense in the marketing literature, showing positive effects (albeit mainly studied in a real world environment). While, scent-enriched VR has already been studied in the context of promoting healthy eating, treating post-traumatic stress disorders, or relaxation therapy, revealing promising (yet not uniform) effects, it’s application for marketing purposes merits further exploration. Flavian et al. (2021) thereby explicitly call for also investigating incongruent scents. Despite being one of the most important aspects of scent, congruity is regarded as an unexplored research area in digital environments (Errajaa et al., 2018).

In a 2 × 3 between-subjects study (*n* = 235), an existing branded VR commercial (Boursin Sensorium Experience) is augmented with “sound” (on/off) and (congruent/incongruent/no) “scents.” The manipulation of scent is informed by the literature on the effectiveness of product-scent congruence, which states that a congruent odor can prime relevant meanings in the consumer’s mind, that in turn facilitate processing of the ad. Such conceptual processing fluency is likely to generate positive feelings that transfer on to the brand that is being advertised, and ultimately benefit advertising effectiveness. The data from this experimental study allow for evaluating these two sensory augmentations (scent and sound) on the basis of emotional, cognitive and conative dimensions of customer engagement. Doing so, this study contributes in answering the following two research questions: (1) How can conventional VR ads be enriched by also addressing the forgotten sense of smell?; and (2) Does doing so indeed instill more engaging experiences?

## Theoretical Background

### Virtual Reality in Marketing

Becoming ever more accessible to consumer markets, Virtual Reality (VR) is currently one of the most promising technologies in terms of business innovation (Goldman Sachs, 2016; Farshid et al., 2018; Hagl and Duane, 2020). Various well-known brands have already jumped on the bandwagon creating virtual brand experiences (Adams, 2016), allowing them to submerge rather passive consumers into an active brand experience (Guppta, 2015). A common goal of VR is to transport users to a virtual environment (VE) and having them experience that environment as though it were real (Martins et al., 2017). The “vividness” of the medium and this feeling of “presence” constitute two major explanatory factors in the effectiveness of VR applications, also in the field of marketing (cf. Loureiro et al., 2021). They have been found to positively affect a number of consumer-related strategically relevant outcomes, such as attitudes toward the medium as well as to the depicted product (Choi and Taylor, 2014; Van Kerrebroeck et al., 2017a); product knowledge (Suh and Chang, 2006); product likability (Verhagen et al., 2014); brand experience (Brakus et al., 2009); brand personality perceptions (De Gauquier et al., 2019); enjoyment (Nah et al., 2011); feelings of relaxation (Serrano et al., 2013); impulse buying (Vonkeman et al., 2017) purchase intentions (Tussyadiah et al., 2017) and mall attitude, satisfaction and loyalty intentions (Van Kerrebroeck et al., 2017b).

While providing a full review of the literature on VR in marketing is beyond the scope of this paper, we refer to a comprehensive text-mining review conducted by Loureiro et al. (2019), in which they cluster and discuss studies according to seven themes for understanding the use of VR in marketing. One of these is particularly relevant as it covers “experiential marketing,” joining studies exploring how VR can improve and transform consumers’ experience. Another relevant group of studies identified covers “communication & social media” and pertains to studies focusing on the use of VR in media communications, social media and advertising. When considering the customer journey, VR can be applied in the “pre-purchase stage,” which covers need recognition and the store/product selection process (e.g., Flavian et al., 2021), the “purchase stage,” which involves the actual purchase process (customer choice, ordering and payment in a VR store, cf. Loureiro et al., 2021) and the “post-purchase stage” which concerns consumption of and engagement with the product/brand and after sales service (cf. Liu et al., 2019; Barbosa Escobar et al., 2021). Also noteworthy is the conceptual framework provided by Hollebeek et al. (2020) on the role of VR in marketing throughout the customer journey, where they distinguish between the pre-, the intra- and the post-VR experience and provide a useful classification of VR archetypes, formats and content features, calling for more research in this domain.

By introducing the EPI Cube, in which technologies are classified according to three human-technology interaction factors: degree of technological embodiment (i.e., external versus internal devices), perceptual presence (i.e., feeling “here” versus “elsewhere”), and level of behavioral interaction (i.e., allowing control & manipulation or not), Flavian et al. (2019) provide a comprehensive overview of how reality-virtuality technologies can affect customer experience in the marketing discipline.

### Digital Sensory Marketing

Human beings’ perception of their environment is strongly influenced by all sensory inputs, including sound, smell, touch and even taste, and by far not just by what can be seen (Calvert et al., 2004). Modality refers to the sensory medium through which information is communicated. Because of their synergetic effect, multi modal cues can be more meaningful than visual-only cues. Therefore, multi-sensory cues can be applied in marketing to affect consumer perceptions, judgments and behaviors (Krishna, 2012). The impact of sensory marketing has been well recognized and examined extensively in physical retail settings (i.e., “store atmospherics,” see Turley and Milliman, 2000; Spence et al., 2014 and Roschk et al., 2017 for extended reviews), as well as in online, mobile and 3D retail settings (i.e., “e-atmospherics,” see Eroglu et al., 2001; “M-atmospherics,” see Rayburn et al., 2022 and “3D-atmospherics,” see Dad et al., 2016 and Krasonikolakis et al., 2018). The power of sensory marketing for advertising purposes has also been clearly established (Krishna et al., 2016). While new technologies are being developed to enable multisensory experiences in a virtual context, opening the path to digital sensory marketing, current digital experiences still remain mainly limited to audiovisual stimulation (Petit et al., 2019). Nevertheless, it is argued by Petit et al. (2019) that appealing to more of the senses should make multisensory online experiences more engaging, immersive, informative, enjoyable and ultimately more persuasive. We refer to Flavian et al. (2021) for a comprehensive overview of multisensory VR research in services, which clearly demonstrates a dearth of empirical studies in a commercial context, but also reveals some ambiguous findings (on the impact of multisensory cues on experienced telepresence for instance), indicating the need for more research within this field.

### The Role of Sound in Augmenting Virtual Reality Ads

By providing additional information, audio-visual cues can enhance visual-only delivered information (Peck and Childers, 2008). Unnava et al. (1996) found that when auditory modality was used in conjunction with visual modality, advertising information was learned better, which can enhance brand associations and attitudes, as well as purchase intentions toward the advertised brand (Wang and Muehling, 2010). In mobile ads the use of audio-visual cues has been shown to lead to more positive perceptions and higher ad and brand recall (Nasco and Bruner, 2007). In advertising music has also been shown to impact ad persuasion by influencing mood and involvement and by conveying meaning (see Krishna et al., 2016 for an overview). When music in an ad works with other elements, such as vision, it strengthens the contextual meaning that is being communicated to the viewer (Hung, 2000), and striking the right cords may move consumers to open their wallets (Strick et al., 2015). Also in retail environments prior research has shown that music can stimulate the senses affecting consumer behavior and influencing their desire to engage in buyer-seller interactions (e.g., Milliman, 1982, 1986; Yalch and Spangenberg, 1990; Dubé et al., 1995; Michel et al., 2017; Dad et al., 2018). In the context of Virtual Reality the importance of sound to augment the sensory experience in order to enhance presence and immersion has been recognized as well (Chandrasekera and Souza, 2015). Serafin and Serafin (2004) for instance have started to examine the role of soundscape design to enhance the sense of presence in virtual reality. Consequently, many resources have been devoted to produce sound effects and ambient sounds that allow the VR user to be fully immersed in the virtual environment (Hohmann et al., 2020). Also for commercial applications the role of sound in VR has received some attention. In the context of a VR shoe store, Loureiro et al. (2021), for instance, recently demonstrated the impact of music tempo, revealing that in contrast to more upbeat music, calm music can leverage the link between presence and intentions to re-visit and recommend the virtual store. On the other hand, playing more upbeat music in the VR shoe store, appeared to establish a stronger link between arousal and pleasure. This indicates that sound is an important element to consider in marketing oriented VR applications and that further research on the subject is warranted.

### The Role of Scent in Augmenting Virtual Reality Ads

Although visual and auditory information are regularly incorporated in virtual environments, the other human senses are often ignored (Ramic-Brkic and Chalmers, 2010). With respect to *scent*-enriched VR experiences, some initial experiments exist, in the context of promoting well-being (e.g., Serrano et al., 2016), healthy eating (Li and Bailenson, 2018), treating alcoholics (Bordnick et al., 2008), or war veterans (Gerardi et al., 2008) and post-traumatic stress disorder patients (Pair et al., 2006). Marketeers are however, steadily becoming aware of the fact that also addressing the “forgotten” senses (i.e., smell, touch, and taste), would allow for more engaging consumer experiences also in a digital context (see Petit et al., 2019 for an overview). While the importance of adding scent to enhance experiences in multisensory digital environments has been recognized (Raisamo et al., 2019), very few studies have examined the application of scents in digital marketing contexts (Serrano et al., 2016; Petit et al., 2019; Roschk and Hosseinpour, 2020; Flavian et al., 2021). Only recently some studies have started examining the value of scent-enriched VR in the context of marketing communications. Flavian et al. (2021), for instance, addressed more specifically the role of “aroma-content congruence” in digital tourist destination experiences and found that adding pleasant and congruent ambient scents to embodied VR experiences impact affective and behavioral reactions directly through sensory stimulation and indirectly via ease of imagination. As the exposure to an unpleasant odor in virtual reality has been shown to increase the sense of presence (Baus and Bouchard, 2017), the question might rise what the impact of an incongruent scent may be (see also Flavian et al., 2021).

### The Role of Product-Scent Congruence in Olfactory Marketing

The main conclusion that can be drawn from the extended literature on olfactory marketing in the physical environment (e.g., store atmosphere) as well as in an advertising context is that *congruence is* key. Congruent scents are scents that are expected in a particular setting because the scent and the setting are thematically matched (Schifferstein and Blok, 2002; Doucé, 2015). Several studies have revealed that scent only has a positive effect when it is congruent with the product category that is sold or advertised (cf. e.g., Mitchell et al., 1995; Bosmans, 2006; Gvili et al., 2018). Fiore et al. (2000) for example found that consumers are more likely to purchase sleepwear, and are moreover willing to pay more, in the presence of a congruent fragrance (as opposed to in the presence of an incongruent smell). Beerli et al. (2021) confirm the positive effects of olfactory marketing on store image, satisfaction and loyalty for an optician store, and particularly highlight that efficacy increases when the congruence between the chosen fragrance and the nature of the store is considered. Gvili et al. (2018) also demonstrate that higher congruence between scent and the advertised product (i.e., liquid soap) heightens positive consumer response. Such congruency effects can be explained by the fact that ambient scents can serve as a prime (e.g., Schab, 1991; Mitchell et al., 1995; Smeets and Dijksterhuis, 2014; Doucé, 2015). Priming can be understood as the incidental perceptual stimulation that enhances the accessibility of concepts that are of use for subsequent information processing (Smeets and Dijksterhuis, 2014). A scent can be an affective prime, meaning that the scent triggers positive consumer reactions (Doucé, 2015), and/or it can be a semantic prime (i.e., cognitive priming; Yi, 1990; Smeets and Dijksterhuis, 2014). The latter implies that when consumers perceive a scent, an automatic knowledge activation process may unconsciously take-off (Mitchell et al., 1995; Schifferstein and Blok, 2002). The scent as such is capable of triggering the activation of stored knowledge, and making certain concepts temporarily more accessible. Such semantic priming can also lead to conceptual fluency, when the information activated by the prime (i.e., the scent) matches with the target element (e.g., an advertised product). Conceptual fluency is a particular type of processing fluency, which refers to the extent to which a consumer experiences ease of processing an external stimulus (Schwarz, 2004). It refers to how readily the stimulus comes to mind and how easily its meaning is understood (Lee and Labroo, 2004). So, when a scent is congruent with the target, it primes target-associated information which enhances the ease of processing the target ad. Previous research on brand choice revealed that conceptual fluency facilitates consideration-set membership and increases brand choice (e.g., Nedungadi, 1990; Lee, 2002). It also improves brand evaluation, since conceptually fluent processing is a positive experience, and this positive affective state can be accredited (incorrectly) to the stimulus, rather than to the ease of processing (Winkielman et al., 2003).

In terms of the effects of incongruent scents, the literature so far produces much more mixed results. Extending the above elaborated line of reasoning on congruence, it could be expected that *incongruent scents* would lead to processing disfluency and cognitive interference, as the information activated by the incongruent scent does not match with the product or the decision task at hand (Doucé, 2015). Incongruent environmental cues have been found to result in lower perceived unity or less coherent ensemble effects (Bell et al., 1991; Mattila and Wirtz, 2001). Scents that are inconsistent with the product have indeed also been found to negatively affect product or ad evaluations (Mitchell et al., 1995; Ellen and Bone, 1998). But there are also findings in favor of the opposite, such as those of Doucé et al. (2016), finding more beneficial effects on customer value perceptions of a retail fashion store, when masculine perfume is diffused in the women’s clothing department (i.e., incongruent product-scent). Spence et al. (2014) moreover indicate that incongruent environments can lead to positive consumer responses because of the “surprise” factor they entail, and the resulting stimulation thereof. This is particularly relevant in specific and unique places, like high-end design stores, like for example a specialty design store selling chairs made from rope (Schifferstein and Spence, 2008). Other studies find no effect of incongruent scent diffusion. Schifferstein and Blok (2002), for example, conclude that an ambient scent of grass does not affect sales of congruent magazines (i.e., nature or football magazines), nor sales of incongruent magazines (i.e., women’s magazines). While this theory has quite extensively been tested already in the context of physical retail environments, it has not yet been examined in the context of VR based (product) brand advertising. Flavian et al. (2021) did investigate the impact of a congruent scent on destination image in a tourism context, finding positive effects, but call explicitly for studies including incongruent scents. Manipulating the congruency of visual, auditory and olfactory cues in a simulated environment using immersive technologies, Liu et al. (2019) explore how these contextual information streams are processed and prioritized by consumers (*N* = 50) and how they affect preference and liking for cold brewed coffee. The incongruent scent used in this study involved the smell of laundry detergent. While the all-incongruent condition led to lower liking scores, this decrease appeared not substantial. This convinces us that further research on the subject is needed.

## Research Objectives and Hypothesis Development

The present study aims to contribute in gaining understanding in how to engage the sense of smell and sound in VR advertising, and whether and how this affects consumer-related strategically relevant outcomes. The particular focus is, besides the typical audiovisual nature of VR, on the sense of *smell* as a key human sense that is often forgotten in marketing in general. Smell adds a richness to our perception and is even capable of altering emotional states (Chu and Downes, 2000; Miles and Jenkins, 2000; Jacob, 2007). Besides its affective impact, the sense of smell also has a substantial diagnostic power. It informs us whether food is safe to eat, when a fire is breaking out in the next room, and even in the evaluation of potential mate attractiveness smell matters (Jacob et al., 2002; Ramic-Brkic and Chalmers, 2010). This study is situated in the pre-purchase stage of the customer journey and has two objectives: (1) identifying what the added value is of augmenting a conventional VR ad with sound and/or scent appeals, taking into consideration product odor congruence, and (2) disentangling the mechanism through which sensory-enriched VR affects customer engagement (CE; Brodie et al., 2011), and ultimately also marketers’ return on investment via sensory enriched VR vividness and telepresence.

### The Effect of Sound and Scent Congruence on Customers’ Sensory Experience of Virtual Reality Ads

Some initial evidence is available in the literature, on how scents can enrich the sensory experience of a VR exposure, to create more compelling, vivid immersions. This evidence is available in a (serious gaming) health context, for purposes of treating obesity, alcohol addiction, PTSD, or merely for instilling full relaxation in people (Serrano et al., 2016). Adding olfaction to a typically merely audiovisual VR experience, has been found to contribute greatly to a sense of presence (Nakamoto and Yoshikawa, 2006; Jung et al., 2020). In a tourism context adding scent to a VR experience has been found to provide more captivating digital destination experiences (Flavian et al., 2021). Drawing from these effects in other applications, it can be expected that *adding a scent* to a VR product advertisement will result in a more sensory rich experience for consumers.

Based on the theoretical underpinnings provided in Section “The Role of Product-Scent Congruence in Olfactory Marketing,” supporting the favorability of selecting *product-congruent scents*, it is expected that this beneficial effect of adding scents to a VR ad will be particularly valid in case the selected scent is congruent with the advertised product (cf. Gvili et al., 2018). Augmenting an ad for cream cheese with a smell of cheese or herbs is likely to function as an odor prime, that activates stored knowledge on the product category, and makes the processing of the information in the VR ad more fluent. The positive affective state resulting from such conceptual (or semantic/thematic) processing fluency, is likely to also favor consumers’ evaluations of the depicted products in the ad. This is in line with the extended literature on congruence effects in marketing (cf. Eklund and Helmefalk, 2021 for an extended overview), and in advertising more specifically, where ad context congruence has also been found to favorably influence people’s reactions toward the ad (cf. van Reijmersdal et al., 2010; Janssens et al., 2012; Kononova and Yuan, 2015; Belanche et al., 2017; Germelmann et al., 2020). Based on the principle of priming (e.g., Yi, 1993), the ad context is generally postulated to prime or activate information that influences ad processing and evaluation, which causes positive ad and brand effects in the case of a congruent ad context (Shen and Chen, 2007). The spreading activation model (Collins and Loftus, 1975; Fazio, 2001) is generally referred at to clarify how the presentation of a prime activates related concepts in a semantic network, which can facilitate ad information processing when relevant knowledge structures have been activated. The positive feeling experienced due to processing fluency and the confirmation of expectations, subsequently have been theorized and demonstrated to transfer to the ad and the advertised product or brand (Mandler, 1982; Sengupta et al., 1997; Campbell and Goodstein, 2001; Dahlen, 2005; Janssens et al., 2012; Lwin et al., 2015; Germelmann et al., 2020).

In terms of product-incongruent scents, the expectation is that consumers will experience such a sensory enrichment as cognitively interfering, complicating their process of getting a grasp of the advertisement in its entirety. If a smell is diffused that is totally irrelated to the product category, processing disfluency is likely to result, with negative effects on consumers’ evaluation toward the advertised product or brand (cf. Gvili et al., 2018). Whereas incongruity between an ad and its context, is sometimes advocated in the literature on advertising effectiveness, because it can make the ad stand out, make it more noticeable and unique, as elucidated by contrast effect theory (Meyers-Levy and Tybout, 1989; Brengman et al., 2001; Dahlen et al., 2008; Vashisht, 2021), it is generally also acknowledged that such incongruity may cause confusion, frustration and dislike. While incongruent ads may be better remembered and recalled as they draw attention and lead to more cognitive elaboration to resolve the incongruity (Heckler and Childers, 1992; Lee and Schumann, 2004) and therefore sometimes be more effective (see Fleck and Maille, 2010 for an extended discussion), they are generally less appreciated (Garbarino and Edell, 1997; Janssens et al., 2012) as schema incongruency inhibits fluent automatic processing and may even evoke a negative conscious persuasion knowledge process caused by perceived manipulative intent, which leads to negative evaluations (Germelmann et al., 2020).

Combined, this leads to the following hypotheses:

H1: The sensory experience customers derive from a VR ad are affected by the distributed *scent*
  H1a: VR ads with *congruent* scent offer a more positive sensory experience than VR ads *without* scent
  H1b: VR ads with *congruent* scent offer a more positive sensory experience than VR ads with *incongruent* scent
  H1c: VR ads *without* scent offer a more positive sensory experience than VR ads with an *incongruent* scent

Because of their synergetic effect, multi-modal cues, such as audio-visual cues, can be expected to be more meaningful and sensory rich than visual-only cues (cf. Spence et al., 2014; Section “Digital Sensory Marketing” above). As such, and in line with the previous argumentations regarding enhanced processing fluency, we expect that adding a pleasant and matching soundscape in a VR ad will positively affect the sensory experience.

H2: VR ads offer customers a more positive sensory experience when including *sound* (as compared to without sound)

Furthermore, this study extends the notion of Gestalt to consumers’ perceptions of *virtual* environments, assuming that – as with the vastly studied domain of *physical* servicescapes (e.g., Spence et al., 2014; Roschk et al., 2017) - consumers perceive such VE’s holistically (cf. Ballantine et al., 2015). In particular, the expectation is that when a VR brand ad offers auditory cues as well as a product-congruent scent, these two sensory stimuli will not work in isolation, but rather strengthen each other’s effect, on the overall sensory experience of the consumer, via enhanced feelings of valence and arousal (cf. Helmefalk and Hultén, 2017). This assumption is in accordance with the extensive review by Spence et al. (2014) who conclude that store atmospherics are evaluated by consumers as more pleasant and interesting when more senses are stimulated and when there is more sensory congruence. Also in the context of marketing communications, it has been established that scent and other sensory cues have a joint effect on consumer behavior (e.g., Estelami and McDonnell, 2007; Gvili et al., 2018). In previous literature the combination of scent and music has been demonstrated to have a positive joint effect on store evaluation and image, product and brand evaluation, purchase intention and satisfaction, time and money spent, and emotional responses (cf. Mattila and Wirtz, 2001; Estelami and McDonnell, 2007; Vaccaro et al., 2009; Morrison et al., 2011; Helmefalk and Hultén, 2017). This is furthermore in line with previous research on VR which has convincingly shown that increasing the number of senses stimulated in a VR environment can dramatically enhance a user’s “sense of presence,” their enjoyment, and even their memory of the experience (Dinh et al., 1999; Gallace et al., 2012). Therefore we also expect a strengthened effect on the sensory experience when both the modality of audition and olfaction are appealed to in VR advertising and formulate the following hypothesis:

H3: There is a positive interaction effect between sound and scent congruence, on the sensory experience of customers

### The Effect of Customers’ Sensory Experience of a Virtual Reality Ad on Customer Engagement

**FIGURE 1:**
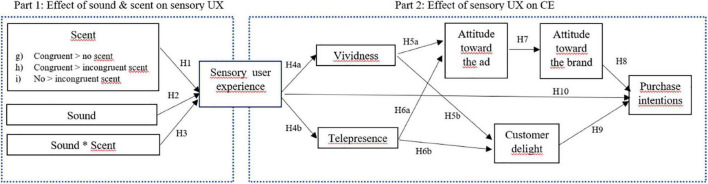
Conceptual model overview.

After hypothesizing direct effects of sound- and scent-manipulations on consumers’ sensory experience, this section formulates expectations regarding how these sensory-enriched experiences translate to higher levels of customer engagement via two vital concepts in VR, namely vividness and (tele)presence. More specifically, a theoretical basis is proposed for understanding how a better sensory VR experience can be expected to ultimately translate into higher purchase intentions (i.e., conative intention), via a cognitive (i.e., brand attitude) and an affective (i.e., customer delight) route, via perceptions of vividness and (tele)presence elicited by the sensory VR experience. Figure 1 provides an overview of our research hypotheses. Note that all hypotheses correspond to expectedly *positive* relationships, based on the existing literature.

Vividness and telepresence actually represent two key concepts in VR. The virtual environment’s degree of “*vividness”* refers to “the representational richness of a mediated environment” (Steuer, 1992, p. 81). The more human senses are engaged in a virtual environment, the more vivid the virtual environment (Fortin and Dholakia, 2005) and the more immersive the experience is perceived to be (Dinh et al., 1999; Feng et al., 2016; Van Kerrebroeck et al., 2017a). The concept of *“telepresence”* refers to the sensation of actually being in the virtual environment and in the depicted scenario (Biocca, 1997; Li et al., 2002). A more sensory stimulating and immersive VR experience is found to evoke stronger feelings of (tele)presence (Gallace et al., 2012; Martins et al., 2017; Jung et al., 2020). However, it should be noted that empirical findings on this are somewhat ambiguous as Baus et al. (2019) and Hopf et al. (2020) did not find presence enhanced in multisensory VR experiences. Still, relying on these conceptualizations we expect that the sensory experience generated by multi-modal VR in a transformational brand communication (VR ad) context will positively impact vividness and telepresence and therefore we suggest the following hypothesis:

H4: A customer’s sensory experience of a VR ad has a positive effect on (a) perceived vividness of the VR ad and (b) perceived telepresence related to the VR ad.

The impact of VR advertising can be ascertained from cognitive, affective, and conative dimensions (Lutz, 1975; Wright, 1980; Hutchinson and Alba, 1991). As with traditional advertising, it is logical to measure the effectiveness of novel types of advertising, such as VR advertising, along these same elements (e.g., Li et al., 2002). Therefore we subsequently link the VR related concepts of vividness and telepresence to these well-accepted ad effectiveness constructs, which include attitude toward the ad and the brand (i.e.. the cognitive route) and customer delight (i.e.. the affective route), which in turn are recognized to have an impact on purchase intentions (i.e.. the conative dimension).

The more *cognitive effect of vividness* on customer attitudes toward the ad has been established and demonstrated by several researchers investigating the impact of media richness (Fortin and Dholakia, 2005). For instance by Klein (2003) when they compared the impact of static (i.e., still pictures and text) versus non-static (i.e., video and audio) informational ads for wine and face cream. In commercial websites vividness was also found associated with more positive and more enduring attitudes toward the website and the advertising and to elicit a positive effect on brand attitude, stimulating consumers’ purchase intentions (Cauberghe et al., 2011). Comparing a VR transformational brand ad video with a 2D version of the ad for an outdoor sports gear brand, Van Kerrebroeck et al. (2017a) also illustrated the positive impact of vividness on attitude toward the ad and on subsequent brand attitude and purchase intentions. See also the meta-analysis of Blondé and Girandola (2016) on the impact of vividness on persuasion. Therefore we formulate the following hypothesis:

H5a: Perceived vividness has a positive effect on customer’s attitude toward the ad

In terms of the *emotional effect of vividness*, it has been argued and proven that vividness can cause enjoyment. For example investigating an augmented reality application, Yim et al. (2017) found a relation between vividness and enjoyment, which was mediated by immersion. Research in the field of immersive movie experiences, has also demonstrated that the emotions viewers experience upon seeing a movie are intensified when viewing it in a more vivid manner [i.e., CAVE versus 2D-viewing, in the experiment by Visch et al. (2010)]. This leads to the following hypothesis:

H5b: Perceived vividness has a positive effect on customer delight

Also for *telepresence* a more *cognitive effect* on customer attitudes toward the ad is expected in accordance to previous conceptualizations. Hopkins et al. (2004) for instance illustrate that perceived telepresence significantly affects consumer responses to online advertising, such as attitude toward the ad, attitude toward the brand and purchase intentions. Also for advergames Sukoco and Wu (2011) found support for a link between telepresence and ad and brand attitudes. Previous research pointed out that to enhance the persuasiveness of VR in the context of tourism and destination marketing, it is imperative to heighten the sense of presence (Tussyadiah et al., 2016). Recent research in the context of web-based and virtual world shopping also confirmed that “being there” matters in influencing consumers’ attitudes (Baker et al., 2019). Therefore, we propose the following hypothesis:

H6a: Perceived telepresence has a positive effect on customer’s attitude toward the ad

The *emotional effect of telepresence* has been well-recognized and established as well (Sylaiou et al., 2010; Nah et al., 2011) and has been argued to be caused by a state of flow evoked by the feeling of actually being in the environment (Faiola et al., 2013; Cheng et al., 2014). Flow refers to the state of mind in which one is no longer aware of one’s real surroundings, which is a consequence of the perception of telepresence (Cho et al., 2002; Nah et al., 2011). In a study on the impact of representation media (2D versus VR) on customer engagement in tourism marketing, for example, Willems et al. (2019) demonstrated the positive impact of telepresence on flow and enjoyment. Therefore we formulate the following hypothesis:

H6b: Perceived telepresence has a positive effect on customer delight

Finally, as commonly acknowledged and explained by the Affect Transfer Theory (Homer, 1990), and also already reported in previously mentioned studies, it can be expected that a positive consumer attitude toward the VR ad transfers on to their attitude toward the advertised brand. In line with the well-established hierarchical model of advertising effects, ad evoked feelings are expected to affect consumer attitudes toward the ad and the brand (Holbrook and Batra, 1987; Pham et al., 2013).

H7: The customer’s attitude toward the ad has a positive effect on the customer’s attitude toward the advertised brand

Consumers’ purchase decision making processes are generally considered to be informed by both ratio and emotion. The more positive consumers think and feel about a certain product or service, the higher their purchase intentions. While a consumer’s attitude toward the brand, is rather cognitive in nature, customer delight can be considered an emotional concept. Both are expected to be positively related to purchase intentions, which is one of the most widely studied conative components of advertising effectiveness (Andrews et al., 1992; Beerli and Santana, 1999). According to Lavidge and Steiner (1961) conation represents the link that drives cognition and affection to behavior. Likewise, Bagozzi (1992) claims that conation contributes to explaining how cognition and emotion are translated into behavior. This basic insight from the field of consumer behavior/psychology and advertising literature, informs the following hypotheses.

H8: The customer’s attitude toward the advertised brand has a positive effect on purchase intentions
H9: Customer delight has a positive effect on purchase intentions

Previous studies have demonstrated that *a VR ad* can have a direct and positive effect on consumers’ intentions toward purchasing an advertised product (e.g., outdoor gear of The North Face; Van Kerrebroeck et al., 2017a) or service (e.g., tourist destination; Willems et al., 2019). Besides the power of VR brand communications to enhance purchase intentions, the *sensory dimension* of any type of customer experience (i.e., in a physical or digital environment) has also been related to increases in consumers’ inclination to purchase (e.g., Lemon and Verhoef, 2016; Moreira et al., 2017; Flavian et al., 2019; Martínez-Navarro et al., 2019). Therefore we expect that the sensory experience of a sensory enriched VR ad will have a direct positive effect on purchase intentions.

H10: A customer’s sensory experience of a sensory enriched VR ad has a direct positive effect on purchase intentions.

## Research Methodology

### Sample and Procedure

A between-subjects 2 × 3 factorial design experiment was conducted, manipulating the senses of sound (on/off) and scent (incongruent/no/congruent) in a VR ad, resulting in the following 6 conditions: (1) VR ad without sound and odor (i.e., baseline or control condition); (2) VR ad with sound but without odor; (3) VR ad with sound and a congruent odor; (4) VR ad with sound and an incongruent odor; (5) VR ad without sound and an incongruent odor; and (6) VR ad without sound and with a congruent odor.

Data on the impact of the respective VR ads was obtained from a student sample of 235 participants (range: 17–29 years old; m_age_ = 21.39 years old, SD = 2.33; 51.1% female), which were randomly distributed over the 6 conditions, taking into account gender quota per condition to guarantee an even spread. Thus, the number of respondents per condition ranged from 37 to 40 participants.

Before exposure to the VR ad a series of starter questions was administered allowing for verification of homogeneity of the respondents across the 6 conditions in terms of potentially confounding variables. Subsequently, the participants were each exposed to only one condition of the experimental stimulus. Finally, a set of post-exposure measures were administered to allow for effect testing.

Regarding the subsamples, an even spread is confirmed across the 6 experimental conditions in gender distribution [χ^2^ (5) = 1.163; *p* > 0.05]. Also, for several control variables no statistically significant differences could be revealed across the conditions, guaranteeing equal subsamples, for example, in terms of prior “brand familiarity” [i.e., knowing the Boursin cheese brand; χ^2^ (5) = 8.656; *p* > 0.05]. This concept was measured by means of 3 answer categories: (1) “yes, I know the brand,” (2) “I have heard about the brand but am not very familiar with it,” and (3) “no.” Also, the proportion of respondents that reported having tasted the brand before [yes/no; χ^2^ (5) = 5.930; *p* > 0.05] did not vary per condition. Neither did the proportion of participants having bought the brand before [yes/no; χ^2^ (5) = 5.691; *p* > 0.05]. Also, the pre-existing attitude toward the Boursin cheese brand proved to be consistent across the subsamples [*F* (5, 234) = 1.472; *p* > 0.05]. This concept was operationalized by means of a summated scale average of 3 Likert-type 7-point scaled items (Homer, 1990), with a very good internal consistency as evidenced by the Cronbach’s α-value of 0.914. Overall, the mean pre-existing brand attitude equaled 5.00 (SD = 0.99), measured on a 1–7 scale. Finally, pre-existing purchase intentions can also be considered similar across the experimental conditions [*F* (5, 234) = 0.440; *p* > 0.05]. For this parameter, the overall mean value equals 4.21 (SD = 1.70).

### Stimuli

The participants were exposed to the “Boursin Sensorium 360 Virtual Reality Experience”^1^, using a smartphone-enabled Google Cardboard-like head-mounted VR device. This marketing communications campaign promoting the cream cheese brand “Boursin” was awarded with several prizes, such as Masters of Marketing Award and the Event Technology Award (Jekimov, 2017). The campaign was developed by a collaboration between Because Experimental Marketing and Hammerhead Interactive Ltd. based on the Oculus Development Kit (Jekimov, 2017). According to the classification by Hollebeek et al. (2020) it can be considered as a VR-centric programmatic VR video providing an experiential narrative for promotional purposes. In this intra-VR experience, the participants find themselves in a virtual rollercoaster that tours throughout a filled fridge. During the ride, the participants are confronted with diverse fruits, vegetables, herbs, bottles and jars, which bring them into contact with complementary tastes and products that match the different Boursin cream cheeses on offer. The total experience lasts about 2 min and was completely new for participants.

A research assistant monitored the experiment conducted early 2018 in a Brussels’ classroom setting and enabled sound (when needed) and provided the necessary olfactory cues (when needed) as soon as the participant had put on the VR headset, in order not to give away the manipulation. After being exposed to the stimulus, the participant was asked to complete a Qualtrics-enabled survey via a standard laptop interface. Upon switching between experimental conditions, the room was ventilated to prevent spill from one odor to a condition with another (or no) odor manipulation.

Upon manipulating the sense of *audition*, the VR ad was either accompanied with the sound as default available in the Boursin Sensorium Experience (see text footnote 1), or without sound. The default Boursin Sensorium soundscape was evaluated during the experiment as rather pleasant (M = 4.99, SD = 1.10) and congruent with the overall experience (M = 4.76, SD = 1.20), whereby both average scores are statistically significantly different from the scale midpoint “4.”

To stimulate the sense of *olfaction*, there were three alternatives: (1) rosemary (= congruent), (2) coffee (= incongruent), or (3) no smell. The research assistant held a box with some of the fresh herb rosemary or freshly grounded coffee under the participant’s nose, as soon as he/she had put on the VR headset. The choice of these particular scents was determined based on a small pre-test among 10 students (7 male, 3 female; average age 22.5 years old) in which a series of 15 food-related scents (of products depicted in the ad and/or used in previous empirical studies on scent effects found in the literature) were generally evaluated by means of a within-subjects experimental design. Both scents were rated similarly in terms of *pleasantness* (M_rosemary_ = 4,85; M_coffee_ = 4,92) and *arousal* (M_rosemary_ = 3,33; M_coffee_ = 3,80), but differed considerably in reported *congruence* (M_rosemary_ = 4,20; M_coffee_ = 1,10) with the Boursin cheese products, which was examined only in a second part of the pre-test in order not to convey this in advance. The latter was confirmed in the actual VR ad experiment, measuring congruence also only at the very end of the questionnaire in order not to draw attention to the odor and convey the purpose of our study. Congruence was operationalized by means of three 7-point Likert scale statements (i.e., “The smell I perceived when watching the VR video, was consistent with the content of the video,” “It is clear that the scent was related to the content of the ad,” and “I think the scent fits with the products of Boursin”; α = 0.92). This corroborated that in terms of *congruence* with the Boursin cheese products the rosemary scent (M = 5.26; SD = 1.28) significantly outperformed the coffee scent [M = 2.5; SD = 1.11; *F*(1, 130) = 191.46; *p* < 0.01].

### Measures

After completing questions gauging for initial brand attitude (3 items, α = 0.91) and purchase intentions (2 items, Pearson’s *r* = 0.92, *p* < 0.01), participants were exposed to one of the six VR conditions. Subsequently, they were asked to fill-out a questionnaire, measuring constructs such as: (1) sensory experience (3 items, α = 0.87), (2) vividness (6 items, α = 0.77), (3) telepresence (8 items, α = 0.80), (4) customer delight (10 items, α = 0.92), (5) attitude toward the brand (3 items, α = 0.92), (6) attitude toward the ad (6 items, α = 0.88), as well as (7) purchase intentions (2 items, Pearson’s *r* = 0.92, *p* < 0.01).

All operationalizations are based on existing, validated scales from the literature, and consist of multi-item scales. The measurement instruments included 7-point Likert scales with anchor points “1” referring to “totally disagree,” to “7” indicating “totally agree” for constructs (pre- and post-) purchase intentions, customer delight, sensory experience, telepresence; and 7-point semantic differential scales for constructs (pre- and post-) brand attitudes, attitude toward the ad, and vividness. The number of items ranged from 2 (for purchase intentions), to 10 (for customer delight). Reliability (e.g., Cronbach’s alpha) and validity (e.g., discriminant validity, HTMT) of all construct operationalizations have been approved of upon evaluating the measurement model (cf. Section “Analysis of the Measurement Model”). The full set of measures can be consulted in Table 1.

**TABLE 1:** Standard loadings, composite reliability and average variance extracted.

Constructs and measured items	Standard loadings
**Sensory experience** (Cronbach’s α = 0.87; CR = 0.94; AVE = 0.92) (adapted from Brakus et al., 2009)	

This VR experience excites the senses	0.89
This VR experience feels like a real sensory experience	0.88
This VR experience stimulates the senses	0.91

**Vividness** (Cronbach’s α = 0.70; CR = 0.81; AVE = 0.81) (Keller and Block, 1997)	

Not vivid – Vivid	0.86
Not easy to imagine – Easy to imagine	0.61
Not easy to relate to – Easy to relate to	0.88
Not easy to picture – Easy to picture	0.89
*Not personal – Personal*	0.85
*Not concrete – Concrete*	

**Telepresence** (Cronbach’s α = 0.85; CR = 0.89; AVE = 0.89) (adapted from Coyle and Thorson, 2001)	

After watching the VR video, I felt like I came back to the “real world” after a journey	0.82
The video created a new world for me, and this new word suddenly disappeared when I took off the VR headset	0.87
While I was watching the VR video, I felt like I was part of the world which Boursin had created	0.70
While I was watching the VR video, I sometimes forgot I was in the middle of an experiment	0.72
While watching the VR video, my body was in the room but my mind was inside the world created by Boursin	0.78
While I was watching the VR video, the environment generated by Boursin was more real or present for me compared to the “real world”	0.63
*The world generated by Boursin seemed to me only “something I saw” rather than “somewhere I visited”*	n.a.
*While I was watching the movie, my mind was in the room, not in the world created by Boursin*	n.a.

**Customer Delight** (Cronbach’s α = 0.92; CR = 0.93; AVE = 0.93) (Oliver et al., 1997)	

Surprised	0.63
Contented	0.79
Happy	0.84
Cheerful	0.84
Pleased	0.80
Excited	0.71
Enthused	0.84
Stimulated	0.76
Elated	0.80
*Astonished*	*n.a.*

**Attitude toward the ad** (Cronbach’s α = 0.88; CR = 0.91; AVE = 0.91) (Madden et al., 1988)	

Unpleasant – pleasant	0.82
Unlikeable – likable	0.86
Tasteless – tasteful	0.76
Boring – interesting	0.72
Artless – artful	0.70
Bad – good	0.87

**Attitude toward the brand** (Cronbach’s α = 0.88; CR = 0.95; AVE = 0.95) (Homer, 1990)	

Positive – negative	0.91
Favorable – unfavorable	0.95
Interesting – uninteresting	0.94

**Purchase intentions** (Cronbach’s α = 0.92; CR = 0.98; AVE = 0.96) (Homer, 1990)	

Likely – unlikely	0.98
Probably – not probable	0.98

**Pleasantness of the smell** (Cronbach’s α = 0.97) (Ellen and Bone, 1998)	

Bad – good	n.a.
Unpleasant – pleasant	n.a.
Negative – positive	n.a.
Smelling bad – smelling good	n.a.

**Scent congruence** (Cronbach’s α = 0.92) (Ellen and Bone, 1998)	

The scent for the advertisement was consistent with what was in the VR video	n.a.
It is clear to me that the scent was related to what was in the ad	n.a.
I think the scent is congruent to Boursin’s products	n.a.

*Items in italics are discarded from the final measurement model, based on cross-loadings, to achieve unidimensionality.*

## Analyses and Results

### The Impact of Sound and Scents on Customer’s Sensory Experience

**FIGURE 2:**
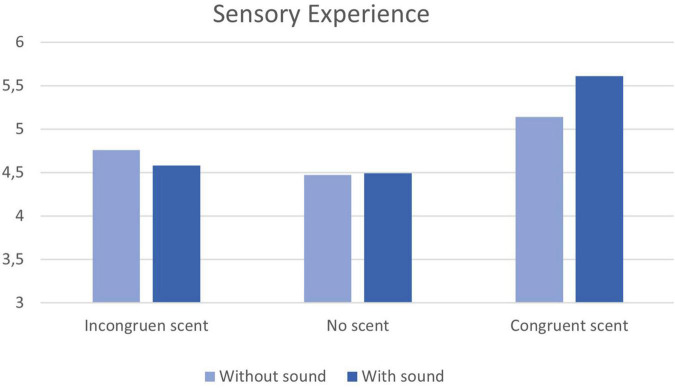
Graphical overview of the mean “sensory experience” in the 6 experimental conditions.

To evaluate the impact of sound and scent on customers’ sensory experience, a univariate analysis of variance is conducted, with *post hoc* multiple pairwise comparisons (LSD). Table 2 provides an overview of the average sensory experience ratings of the respondents for each of the six conditions. Figure 2 provides a graphical summary.

**TABLE 2:** Descriptive statistics “Sensory Experience” across all six experimental conditions.

Condition	Incongruent scent	No scent	Congruent scent
Without sound	Condition (1)	Condition (2)	Condition (3)

	M = 4.76	M = 4.47	M = 5.14
	(SD = 1.06) * (6)	(SD = 1.06) * (3), (6)	(SD = 1.16) * (2), (4), (5)

With sound	Condition (4)	Condition (5)	Condition (6)

	M = 4.58	M = 4.49	M = 5.61
	(SD = 1.32) * (3), (6)	(SD = 1.37) * (3), (6)	(SD = 1.22) * (1), (2), (4) & (5)

Congruent scent = rosemary; incongruent scent = coffee. *Pairwise comparisons, p < 0.05.

First, we address H3 and inspect whether there is a statistically significant interaction effect between the factor “sound” (without sound/with sound) and the factor “scent congruence” (without scent/with congruent scent/with incongruent scent). A 2 × 3 ANOVA reveals that there is no statistically significant interaction effect [F (2, 234) = 1.46, *p* = 0.24 > 0.05].

With respect to sound (H2), we find no significant main effect either [*F* (1, 234) = 0.43, *p* = 0.51 > 0.05]. An examination of the pairwise comparisons reveals that adding sound to the VR ad only seems to increase the average level of sensory experience reported by consumers marginally in the presence of a congruent scent (*p* = 0.09 < 0.10).

With regard to scent we do find a statistically significant main effect [*F* (2, 234) = 11.81, *p* < 0.01]. Addressing H1a specifically, the results confirm that the average sensory experience is significantly higher when the congruent rosemary scent is added to the VR experience, and this both in the absence of sound (M_Rosemary_ = 5.14, SD_Rosemary_ = 1.16 vs. M_No___scent_ = 4.47, SD_No_scent_ = 1.06; *p* = 0.014 < 0.05), as well as in its presence (M_Rosemary_ = 5.61, SD_Rosemary_ = 1.22 vs. M_No___scent_ = 4.49, SD_No___scent_ = 1.37; *p* < 0.001). With respect to H1b, the average sensory experience evoked by the VR with congruent rosemary scent and sound (M = 5.61, SD = 1.22) is statistically significantly higher than that elicited by the VR with incongruent coffee scent and sound (M = 4.58, SD = 1.32; *p* < 0.01). When inspecting the pairwise comparison of rosemary (i.e., congruent) scent versus coffee (i.e., incongruent) scent, in the absence of sound, however, the average sensory experience as rated by the respondents does not differ significantly (M_Rosemary_ = 5.14, SD_Rosemary_ = 1.16 versus M_Coffee_ = 4.76, SD_Coffee_ = 1.06; *p* = 0.17 > 0.05). Finally, testing H1c, the pairwise comparison of the incongruent coffee scent condition with sound (M = 4.58, SD = 1.32) with the no scent condition with sound (M = 4.49, SD = 1.37) indicates no statistically significant difference (*p* = 0.76 > 0.05). Also, in the absence of sound, the two scent conditions do not differ significantly (M_Coffee_ = 4.76, SD_Coffee_ = 1.06 versus M_No_Scent_ = 4.47, SD_No_Scent_ = 1.06; *p* = 0.29 > 0.05).

Summing up, there is support for H1a and (partially for) H1b, but not for H1c, nor for H2 or H3. Apparently, it is mainly a congruent scent that seems to make a difference in the level of sensory experience the VR Boursin Sensorium can instill in customers. An incongruent or no scent, does not seem to make a significant difference, at least when sound is not enabled. However, when sound is also in the picture, adding a congruent scent does generate statistically significantly more positive sensory experiences on average, as compared to no scent or an incongruent scent. To conclude, the congruent scent outperforms the no scent conditions, regardless whether sound is enabled or not. However, as to the incongruent scent, the results show that the congruent scent is only significantly better, when sound is also enabled.

### The Strategic Role of Customers’ Sensory Experience on Customer Engagement

To test the remainder of our research model (i.e., H4-H11), we conducted Partial Least Squares Path Modeling (PLS-PM) to analyze the relationships between the constructs in the conceptual model.

#### Analysis of the Measurement Model

First, the measurement model was evaluated. All latent constructs in this model contain reflective items for which unidimensionality is tested. Unidimensionality refers to the existence of a single construct underlying a set of indicators. Besides an inspection of internal consistency of the items underlying each latent construct (cf. Cronbach’s alpha’s and Pearson correlation coefficients, as reported in Section “Measures”), further examination relies on Karlis et al. (2003)’s procedure. A set of items can be considered unidimensional if the first eigenvalue of the correlation matrix of items exceeds one, and the second value is smaller than one. The procedure by Karlis et al. (2003) confirms that the conditions are met for all reflective constructs in the model apart from “Customer delight,” “Vividness,” and “Telepresence.” For these constructs, respectively 1, 2, and 2 items are deleted, in order to achieve a unidimensional multi-item measure of the respective underlying constructs. In Table 1, these items are marked in italics. Doing so, all constructs can be considered as unidimensional. The resulting Cronbach’s alpha value for “Customer delight” (9 items) becomes 0.92; for “Vividness” (4 items) it is 0.71; and for “Telepresence” (6 items) the value is 0.85.

Next, further psychometric property testing of the measurement model includes checking item validity and discriminant validity. The PLS analyses indicated high factor loadings for most items (cf. Table 1), namely reaching or exceeding the 0.70 level. A bootstrapping procedure with 5000 resamples moreover confirms the statistical significance of every item loading. While 2 item loadings (pertaining to the latent variable “telepresence” and respectively “customer delight”) equaled 0.63 (i.e., below the threshold of 0.70), we chose to retain them as it is considered appropriate to keep the items to avoid losing relevant content (Barclay et al., 1995). Discriminant validity was furthermore assessed by comparing the square root of the average variance extracted (AVE) with the correlations between the constructs (i.e., the Fornell and Larcker, 1981 criterion). Every diagonal value is found to be higher than the off-diagonal values in its column, thus indicating that discriminant validity is established (Hair et al., 2017; cf. Table 3). Further screening of the Heterotrait-monotrait ratio (HTMT) of the correlations revealed that they range from 0.26 to 0.69, with as such none of the HTMT values exceeding the threshold value of 0.90 (Henseler et al., 2015), providing further assurance of discriminant validity in our resulting final measurement model.

**TABLE 3:** Descriptive statistics and latent variable correlation matrix: discriminant validity (*n* = 669).

	1	2	3	4	5	6	7
1. Attitude toward the ad	**0.80**						
2. Attitude toward the brand	0.39	**0.93**					
3. Customer delight	0.63	0.49	**0.78**				
4. Purchase intentions	0.25	0.58	0.40	**0.98**			
5. Sensory user experience	0.61	0.26	0.61	0.23	**0.90**		
6. Telepresence	0.46	0.27	0.58	0.33	0.56	**0.76**	
7. Vividness	0.61	0.30	0.56	0.29	0.48	0.48	**0.72**

*Bold numbers on the diagonal show the square root of AVE.*

#### Analysis of the Hypothesized Structural Relationships in the Model

**FIGURE 3:**
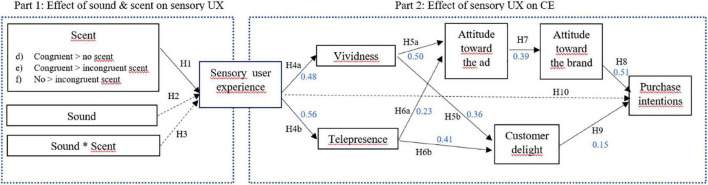
Overview of the findings.

PLS-PM and a 5000-resample bootstrap was applied to analyze the relationships indicated in the conceptual model and to evaluate its statistical significance. PLS is a variance-based estimation technique which is not restrictive in terms of dataset distribution and can handle more complex models (Hair et al., 2017). Note that in the remainder of this section, *p*-values are provided accompanying all path coefficients under study. The inferences drawn on the basis of bias-corrected confidence intervals are completely in line. The overall goodness-of-fit is evaluated on the basis of the coefficient of determination. With an adjusted R^2^ value of 0.35, for the endogenous variable “Purchase intentions,” the predictive power of the model can be considered moderate (Chin, 1998; Hair et al., 2017). Furthermore, Q^2^ value can be considered as medium for the core endogenous variable under study (i.e., Purchase intentions, Q^2^ = 0.32; Chin, 1998). The SRMR demonstrates that the model has a good fit (SRMR = 0.069; *p* < 0.001) (Hu and Bentler, 1998; Henseler et al., 2014). As such, the structural model analysis can proceed with an examination of the path coefficients. See Figure 3.

First, customers’ sensory experience evoked by the Boursin Sensorium VR experience is found to have a statistically significant positive effect on both vividness (H4a; β = 0.48; *p* < 0.01), as well as telepresence (H4b; β = 0.56; *p* < 0.01). Second, we hypothesized that perceived vividness (H5) and telepresence (H6) in the VR experience have a cognitive as well as an affective effect on consumers. For vividness, the effect mainly runs in a cognitive way, having a stronger impact on consumers attitude toward the ad (i.e., H5a, β = 0.50; *p* < 0.01) than on feelings of customer delight (H5b, β = 0.36; *p* < 0.01). For telepresence, the findings are the opposite, as feeling more present in the VE rather drives customer delight (H6b, β = 0.41; *p* < 0.01), than their attitudes toward the VR ad (H6a, β = 0.23; *p* < 0.01). As commonly acknowledged (cf. Affect transfer hypothesis; Homer, 1990), we tested nomological network validity by inspecting whether our data also confirm that a positive consumer attitude toward the VR ad transfers on to their attitude toward the advertised brand. We find support for this relationship, put forward in H7 (β = 0.39; *p* < 0.01). Furthermore, customers’ attitude toward the brand (i.e., cognitive route; H8) has a stronger statistically significant positive effect on purchase intentions (β = 0.51; *p* < 0.01), than feelings of customer delight (i.e., affective route; H9, β = 0.15; *p* = 0.03 < 0.05).

As we fail to find support for a direct link between the sensory experience and purchase intentions (H10; β = 0.001; *p* = 0.99 > 0.05), this relationship appears to be mediated and explained by the interlaying variables discussed in H4-H9. According to the procedure for mediation analysis outlined by Zhao et al. (2010), we first establish that the indirect effect is significant. Based on the path coefficients generated by the PLS analysis, the total indirect effect path coefficient, aggregated over the different mediating paths equals 0.14, with confidence interval based on the aforementioned 5000-resample bootstrapping procedure at the 5% level of [0.15; 0.14], which does not include zero and thus proves to be significant (Nitzl et al., 2016). The variance accounted for (VAF; i.e., the size of the indirect effect in relation to the total effect) amounts to 98.52%. Combining the lack of statistical significance of the direct path between sensory user experience and purchase intentions, and this strong indirect effect, this analysis points at full mediation between sensory experience and online purchase intentions via vividness and telepresence, and subsequently the affective (delight) and cognitive (attitude) routes (Hair et al., 2017).

## Conclusion and Discussion

### Discussion

With respect to the effect of sound and scent on the sensory experience of customers, our findings identify a *congruent scent* as a deal maker. Whether sound is enabled or not, adding a product-congruent scent (e.g., rosemary herb for a cream cheese ad), consistently results in a more compelling sensory experience, than without enriching the VR ad with any scent. This is in line with previous research in the context of store atmospherics and advertising (cf. Fiore et al., 2000; Gvili et al., 2018; Beerli et al., 2021) and confirms and extends the findings in the context of VR ads beyond digital tourist destination pre-experiences (cf. Flavian et al., 2021).

Apparently, adding an *incongruent scent* (as opposed to no scent) does however, not seem to be a deal breaker – at least not in terms of the sensory experience. The incongruent scent conditions of this study’s experiment do not result in significantly lower sensory experiences among consumers than the conditions without scent-augmentation. Albeit without statistical significance, the findings show that adding an incongruent smell results even in a slightly better sensory experience than without adding any scent to the VR ad. Note that this finding is not entirely unexpected, given the mixed findings of earlier studies on incongruent product-scents in physical retail environments (cf. Schifferstein and Blok, 2002; Doucé et al., 2016) and incongruent ad-contexts (cf. Fleck and Maille, 2010; Janssens et al., 2012). Likewise, investigating a VR enriched product experience, which can be considered a VR application situated in the “post-purchase stage” of the customer journey (cf. Loureiro et al., 2021), Liu et al. (2019) also did not find the diffusion of an incongruent scent to lead to substantially decreased liking scores.

For the particular manipulation in the present study’s experiment, however, it might be the case that respondents perceive of the coffee (i.e., incongruent scent with Boursin cream cheese) as a smell that they associate with the theme of “breakfast” or “kitchen,” just like they do for cream cheese. While our manipulation check identified rosemary herb smell as congruent with the herby cream cheese, and the coffee smell as incongruent, it might as such be that the two still match under the common denominator of “breakfast” drinks and food. If so, this would imply semantic processing fluency, which could have beneficially impacted the effect of the “incongruent” scent in the experiment.

But even if coffee – being associated with breakfast (like cream cheese can be) – is thematically linked to the general *product category* of the ad, one would expect the scent that is linked to the *particular product* to be more congruent. As such, it would still be likely that the congruent scent condition (i.e., rosemary) outperforms the incongruent (or less congruent) scent condition (i.e., coffee) in terms of the sensory experience it evokes in consumers. The present study’s findings point out that this is only the case (with statistical significance), when sound is also enabled. This finding may be interpreted in light of the theoretical rationale elaborated in Section “The Role of Product-Scent Congruence in Olfactory Marketing.” When sound is enabled, and an incongruent (or less congruent) scent is added, consumers may become cognitively challenged, as the two sensory stimuli interfere, and the consumer has to make sense of it. The incongruent scent, and the presence of sound would be cognitively taxing, and complicating the processing of the ad (cf. Lee and Schumann, 2004 and Germelmann et al., 2020). This is also consistent with findings from previous studies that found incongruent environmental cues to result in lower perceived unity or less coherent ensemble effects (Bell et al., 1991; Mattila and Wirtz, 2001).

This may be one conjecture on the fact that we only find significant results in favor of the congruent scent, when sound is also enabled. This confirms that, also in a VR context, the auditory track in an ad can strengthen the contextual meaning that is being conveyed, but only when there is a fit between the different elements of the ad (cf. Hung, 2000). Partly in accordance with previous research on VR, which has convincingly shown that increasing the number of senses stimulated in a VR environment can dramatically enhance a user’s experience (Dinh et al., 1999; Gallace et al., 2012), this study nuances the picture somewhat, by highlighting the importance of the congruence of ad cues.

This study’s findings clearly illustrate that upon studying sensory-enriched digital consumer experiences (such as a scent-augmented VR ad) – as in the vast literature on the physical servicescape (e.g., Mattila and Wirtz, 2001; Spence et al., 2014; Roschk et al., 2017) - ambient stimuli should not be considered in isolation, since it is the total configuration of cues that influence consumer responses.

This is also supported by the findings of Liu et al. (2019) in a VR experience context enriched with visual, auditory and olfactory contextual cues, where they did not find any impact of the incongruent sensory stimuli individually, but did observe a significant (albeit small) effect on product (coffee) liking in the all incongruent condition.

With respect to *sound*, in the present VR case (Boursin Sensorium Experience), no significant main effect was found, nor did we find a general strengthening effect at play upon stimulating an interplay of audition and olfaction. Only a marginal effect was found of enabling sound in the presence of a congruent scent. The lack of overall proof for the added value of enabling sound in a VR advertisement could be explained by the particular ad portrayed in this experiment, namely an ad for cream cheese. Bruner (1990) conducted studies on the effect of ambient music (in the physical environment) and found that music has the greatest effect on consumer behavior when consumers are highly affectively involved. When buying products that provide psychological benefits (e.g., prestigious clothing), consumers are strongly guided by their emotions. This can explain why, in contrast to our findings, Loureiro et al. (2021) did find significant positive effects of music in a VR shoe store. Cream cheese is however, not a prototypical product that can be considered emotionally involving. For other product categories, sound may as such still offer a significant added value to the consumer’s overall sensory user experience. Furthermore, in this experiment sound has simply been manipulated by turning it on or off. However, there are several different aspects of sound that can be manipulated (e.g., genre, tempo, pitch, tone, etc.). Especially in the context of VR, composing a dynamic soundscape is still quite challenging for sound designers. It would be interesting to test the impact of different more detailed sound manipulations.

Note that the study findings also suggest that stronger sensory experiences do *not directly* drive purchase intentions, but they do increase the audience’s vividness perceptions and their feeling of being present in the virtual environment, both of which in turn influence consumers’ emotions and cognitive evaluations, which ultimately do drive their purchase intentions.

### Managerial Implications

VR is increasingly being appreciated for its power of engaging customers in marketing communications (Boyd and Koles, 2019). Therefore it is important that practitioners understand what makes an effective VR experience. Especially sensory-enriched VR advertising allows to vividly immerse consumers in staged transformational brand experiences, as if they were actually present in the scene (be it at home, in- store, or online), inducing more positive brand attitudes, delight and higher purchase intentions. Particularly when using congruent scents in the VR experience. Advances are made in computer sciences, in developing olfaction-enhanced multimedia applications, that combine computer generated smell with other media to enrich the users’ experience (Ghinea and Ademoye, 2011; Spence et al., 2017; Brooks et al., 2021). Such developments are also relevant to allow for sensory stimulation embedded in VR (e.g., Nambu et al., 2010; Brooks et al., 2020). Once such applications become commercially available, this will further extend the possibilities of VR to be leveraged by marketers, which may especially be relevant for the branding of experience goods, such as food or apparel. The recent investments of Meta (the social media company formerly known as Facebook) in its Metaverse (which is essentially an evolution of the internet consisting of three-dimensional spaces in virtual reality where users can interact with each other) and its Reality Lab developing multisensory VR interfaces, such as haptic gloves, is indicative of the commercial value envisioned for such multisensory VR applications. While Facebook hasn’t claimed to be working on any olfactory-oriented VR products yet, a number of companies are now developing devices that would allow people to “smell” the metaverse (Mak, 2021). However, despite the efforts made by researchers and practitioners to deliver multisensory digital experiences, there is still a long way to go before this goal is accomplished (Petit et al., 2019). Providing multisensory experiences in digital environments has become one of the future priorities in technology development (Velasco and Obrist, 2020).

### Limitations and Suggestions for Further Research

More research is called for to enrich our understanding on how to manipulate auditory cues on the one hand, and whether (and how) to address the remaining “forgotten” senses of tactition and gustation in producing VR marketing appeals.

First, regarding audition, there is need for follow-up research whereby the effect of sound congruence is examined, in line with how the present study inspected the impact of odor congruence in VR advertising. Indeed, auditory features can also be used to convey or accentuate certain product characteristics via semantic congruency (Minsky and Fahey, 2017). In the current experiment there was either the default soundscape available in the Boursin Sensorium Experience (which was experienced as fairly pleasant and congruent), or no sound at all. Future studies could aim at manipulating sound with more extremely congruent and resp. incongruent auditory stimuli, to allow for inspecting their relative effectiveness (cf. Hung, 2000; Hagtvedt and Brasel, 2016; Knoeferle et al., 2016 and Liu et al., 2019). Furthermore, the impact of numerous other aspects of soundscapes, such as tempo, pitch or frequency, volume, intensity, duration, style, sonic seasoning and emotional depth (cf. Strick et al., 2015; Roschk et al., 2017) should be investigated further, especially in dynamic interactive VR environments (Loureiro et al., 2021).

Also for the odor manipulation, we made use of the food related scents rosemary and coffee, which are respectively more or less congruent with the product under study, however, a more outspoken incongruent scent than the smell of coffee could be used. In previous research the smell of coffee has also unexpectedly been found more congruent to certain visual stimuli as intended (cf. Barbosa Escobar et al., 2021). Thorough pre-testing of stimuli before conducting further elaborate experiments is therefore advisable. Likewise some other scent attributes could be considered, such as scent complexity (Herrmann et al., 2013), intensity (Maggioni et al., 2020) or temperature associations (Krishna et al., 2010; Madzharov et al., 2015), which were not specifically taken into account in the current study. The “warm” smell of coffee may for instance have offset the “cool” fridge temperature, causing more positive effects, which is to be examined more in depth in future research.

Second, with regards to whether (and how) to manipulate the other forgotten senses, some scant research findings are published (see Petit et al., 2019 for an overview), like Serrano et al.’s (2016) findings on the tactile sensation of grass under one’s bare feet, upon experiencing a VR meadow/football game, while also smelling grass. In the Boursin Sensorium Experience, one could consider adding a breeze of fresh air to augment the feeling of flowing through the virtual fridge [cf. the “Season Traveler” recently developed by Ranasinghe et al. (2018)]. Likewise, promo girls/boys could offer Boursin tasters, upon providing this sort of multi-sensory VR experience in for example a supermarket or shopping mall. Would this sense of touch and taste further enrich customers’ engagement with the brand? Would it install more enduring brand impressions? These are all questions that can feed follow-up research in this fruitful avenue.

Third, as to methodological advice, it is recommendable that future research also examines the impact of multi-sensory VR advertising among non-student samples. While some studies suggest that this may not significantly affect validity when the research involves individual decision-making (McKnight et al., 2002) and effects of advertising (Singh et al., 2000; Wan et al., 2007), it should be acknowledged that the findings of the study may not be generalizable to other generations of consumers (Mazaheri et al., 2012; Peterson and Merunka, 2014). While we do find some initial support by Gibson and O’Rawe (2018; p. 102) for the absence of a relationship between VR usage/response and age, we recognize that further research to confirm this preliminary finding is recommendable. Further research should examine how an older sample group reacts to innovative media, as younger people are, for instance, often more likely to seek sensation (Xu et al., 2016) and are more open to new technologies (Venkatesh et al., 2003). A study considering a more diverse respondent sample, considering all age groups could allow for the comparison between age groups, possibly identifying age as a moderator.

Additionally, it is recommendable to evaluate alternative options of measuring (a) customer emotions (e.g., neurophysiological techniques, such as pupil dilatation or galvanic skin response analysis; Ramsøy et al., 2017), (b) cognitive appreciation or attitudes (e.g., Implicit Association Testing; IAT; e.g., Greenwald et al., 1998; Dimofte, 2010; Goodall, 2011), and (c) behavior (e.g., by means of observational studies) or (d) other marketing KPIs (e.g., secondary data sources, such as actual sales records). Until present only a minority of survey research in marketing has included data from such sources [i.e., 6.4% of all survey-research based marketing publications in JAMS, JM and JMR, as evidenced in a recent review study of the period 2006–2015, by Hulland et al. (2018)]. Furthermore, field studies are also particularly called for to examine the external validity of our findings.

## Data Availability Statement

The raw data supporting the conclusions of this article will be made available by the authors, without undue reservation.

## Ethics Statement

Ethical review and approval was not required for the study on human participants in accordance with the local legislation and institutional requirements. The patients/participants provided their written informed consent to participate in this study.

## Author Contributions

MB and LD took the lead in designing this study and coordinating the data collection. KW mainly contributed in the analyses and reporting. All authors contributed to the article and approved the submitted version.

## Conflict of Interest

The authors declare that the research was conducted in the absence of any commercial or financial relationships that could be construed as a potential conflict of interest.

## Publisher’s Note

All claims expressed in this article are solely those of the authors and do not necessarily represent those of their affiliated organizations, or those of the publisher, the editors and the reviewers. Any product that may be evaluated in this article, or claim that may be made by its manufacturer, is not guaranteed or endorsed by the publisher.

## Footnotes

1 cf. http://bit.do/e8suL
